# Coffee drinking timing and mortality in US adults

**DOI:** 10.1093/eurheartj/ehae871

**Published:** 2025-01-08

**Authors:** Xuan Wang, Hao Ma, Qi Sun, Jun Li, Yoriko Heianza, Rob M Van Dam, Frank B Hu, Eric Rimm, JoAnn E Manson, Lu Qi

**Affiliations:** Department of Epidemiology, School of Public Health and Tropical Medicine, Tulane University, 1440 Canal Street, New Orleans, LA 70112, USA; Department of Epidemiology, School of Public Health and Tropical Medicine, Tulane University, 1440 Canal Street, New Orleans, LA 70112, USA; Department of Nutrition, Harvard T.H. Chan School of Public Health, 665 Huntington Avenue, Boston, MA 02115, USA; Channing Division of Network Medicine, Department of Medicine, Brigham and Women’s Hospital and Harvard Medical School, Boston, MA, USA; Department of Nutrition, Harvard T.H. Chan School of Public Health, 665 Huntington Avenue, Boston, MA 02115, USA; Department of Epidemiology, Harvard T.H. Chan School of Public Health, Boston, MA, USA; Department of Epidemiology, School of Public Health and Tropical Medicine, Tulane University, 1440 Canal Street, New Orleans, LA 70112, USA; Departments of Exercise and Nutrition Sciences and Epidemiology, Milken Institute School of Public Health, The George Washington University, Washington, DC, USA; Department of Nutrition, Harvard T.H. Chan School of Public Health, 665 Huntington Avenue, Boston, MA 02115, USA; Channing Division of Network Medicine, Department of Medicine, Brigham and Women’s Hospital and Harvard Medical School, Boston, MA, USA; Department of Nutrition, Harvard T.H. Chan School of Public Health, 665 Huntington Avenue, Boston, MA 02115, USA; Channing Division of Network Medicine, Department of Medicine, Brigham and Women’s Hospital and Harvard Medical School, Boston, MA, USA; Department of Epidemiology, Harvard T.H. Chan School of Public Health, Boston, MA, USA; Channing Division of Network Medicine, Department of Medicine, Brigham and Women’s Hospital and Harvard Medical School, Boston, MA, USA; Department of Epidemiology, Harvard T.H. Chan School of Public Health, Boston, MA, USA; Division of Preventive Medicine, Department of Medicine, Brigham and Women’s Hospital, Harvard Medical School, Boston, MA, USA; Department of Epidemiology, School of Public Health and Tropical Medicine, Tulane University, 1440 Canal Street, New Orleans, LA 70112, USA; Department of Nutrition, Harvard T.H. Chan School of Public Health, 665 Huntington Avenue, Boston, MA 02115, USA

**Keywords:** Coffee intake, Timing, CVD mortality, All-cause mortality

## Abstract

**Background and Aims:**

To identify the patterns of coffee drinking timing in the US population and evaluate their associations with all-cause and cause-specific mortality.

**Methods:**

This study included 40 725 adults from the National Health and Nutrition Examination Survey 1999–2018 who had complete information on dietary data and 1463 adults from the Women’s and Men’s Lifestyle Validation Study who had complete data on 7-day dietary record. Clustering analysis was used to identify patterns of coffee drinking timing.

**Results:**

In this observational study, two distinct patterns of coffee drinking timing [morning type (36% of participants) and all-day-type patterns (14% of participants)] were identified in the National Health and Nutrition Examination Survey and were validated in the Women’s and Men’s Lifestyle Validation Study. During a median (interquartile range) follow-up of 9.8 (9.1) years, a total of 4295 all-cause deaths, 1268 cardiovascular disease deaths, and 934 cancer deaths were recorded. After adjustment for caffeinated and decaffeinated coffee intake amounts, sleep hours, and other confounders, the morning-type pattern, rather than the all-day-type pattern, was significantly associated with lower risks of all-cause (hazard ratio: .84; 95% confidential interval: .74–.95) and cardiovascular disease-specific (hazard ratio: .69; 95% confidential interval: .55–.87) mortality as compared with non-coffee drinking. Coffee drinking timing significantly modified the association between coffee intake amounts and all-cause mortality (*P*-interaction = .031); higher coffee intake amounts were significantly associated with a lower risk of all-cause mortality in participants with morning-type pattern but not in those with all-day-type pattern.

**Conclusions:**

Drinking coffee in the morning may be more strongly associated with a lower risk of mortality than drinking coffee later in the day.

## Structured Graphical Abstract

**Structured Graphical Abstract:**
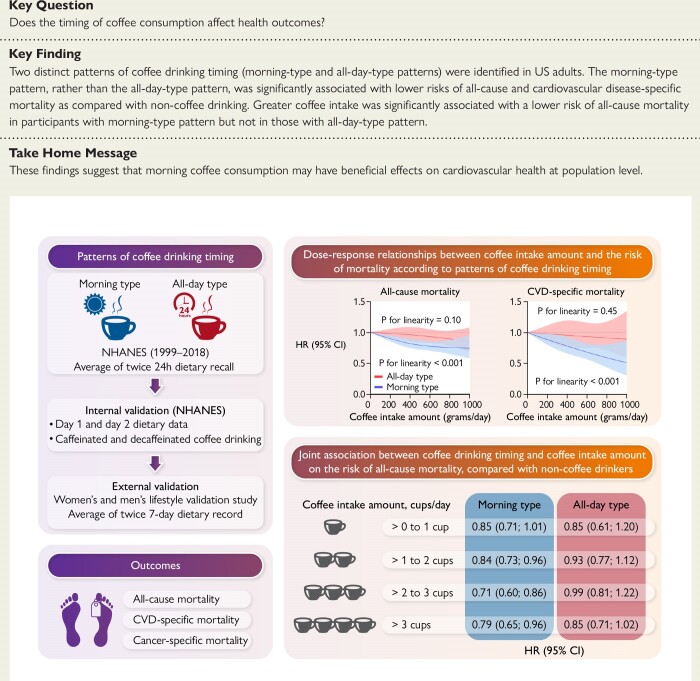
Coffee drinking timing, coffee intake amounts and the risk of mortality. NHANES, National Health and Nutrition Examination Survey; CI, confidential interval; CVD, cardiovascular disease; HR, hazard ratio.

**See the editorial comment for this article ‘Start your day with a morning coffee!’, by T.F. Lüscher, https://doi.org/10.1093/eurheartj/ehae823.**

## Introduction

Coffee is one of the most commonly consumed beverages in the world.^1^ Most prospective studies have found that moderate coffee consumption is associated with lower risks of Type 2 diabetes, cardiovascular diseases (CVDs), and death.^2,3^ The 2015–20 US Dietary Guidelines recommend moderate coffee consumption as part of a healthy dietary pattern.^4^ However, the association between heavy coffee consumption (more than 3–5 cups/day) and mortality risk is still controversial.^3,5–7^ Given the importance of coffee to daily life, several studies have explored whether clinical or behavioural factors may modify the association between coffee intake, especially heavy coffee intake, and health outcomes, including smoking,^5,6^ decaffeinated coffee intake,^5^ genetically determined caffeine metabolism rate,^6,8,9^ and sweeteners added to coffee,^10,11^ as well as the coffee brewing method.^12^ However, no consistent evidence supports these factors modify the association of coffee intake with health outcomes.

Notably, increasing evidence has indicated the importance of circadian rhythm in regulating human food intake behaviours and metabolism,^13^ and some previous studies have shown that the timing of food intake may modify the association of food intake with health outcome.^14–17^ Intriguingly, coffee has long been used to improve wakefulness and relieve drowsiness due to the stimulating effects of caffeine on the central nervous system.^18,19^ However, drinking coffee later in the day may disrupt the daily circadian rhythms and thus modify the association between the amount of coffee intake and health outcomes.^20^ It is unclear whether there are distinct patterns of coffee drinking timing in the population, and if so, whether these patterns are associated with mortality risk.

In this study, we identified patterns of coffee drinking timing in the population through cluster analysis in the National Health and Nutrition Examination Survey (NHANES) and validated these patterns in the Women’s and Men’s Lifestyle Validation Study (WLVS and MLVS), two sub-studies conducted in the Nurse’s Health Study (NHS) and NHS II, and the Health Professionals Follow-up Study (HPFS) cohort. Subsequently, we investigated the joint association between patterns of coffee drinking timing and amounts of coffee intake and the risk of all-cause and cause-specific mortality in NHANES.

## Methods

### Study design and population

This study included participants from a prospective cohort in the NHANES to identify patterns of coffee drinking timing and relate these to mortality. We also included participants from WLVS and MLVS (two sub-studies to validate lifestyle assessments for the NHS and NHS II, and HPFS) to externally validate the patterns of coffee drinking timing identified in NHANES. National Health and Nutrition Examination Survey is a nationally representative study conducted by the National Center for Health Statistics of the Centers for Disease Control and Prevention to assess the health and nutritional status of adults and children in the United States. The study design and methods have been described in detail previously (www.cdc.gov/nchs/nhanes/about_nhanes.htm.). The survey protocol was approved by the Research Ethics Review Board of the National Center for Health Statistics. Written informed consent from all participants was obtained by NHANES. This cohort study included adults 18 years of age or older who provided available dietary recall data and mortality data during 10 NHANES cycles from 1999–2000 to 2017–18. A total of 40 725 participants were eligible for this study (see Supplementary data online, *Figure S1*), after excluding participants who had self-reported CVD or cancer at baseline (*n* = 8077), who were pregnant at the time of examination (*n* = 1478) or had implausible total energy intake (<500 or >3500 kcal/day for women and <800 or >4200 kcal/day for men; *n* = 2105).

The WLVS was conducted from 2010 to 2012 and consisted of 796 women aged 45–80 years randomly sampled from the NHS and NHS II, two ongoing, nationwide, prospective cohorts of US female registered nurses established in 1976 and 1989, respectively.^21–23^ The MLVS was conducted from 2011 to 2013 and consisted of 914 men aged 46–82 years randomly recruited from the HPFS and members of Harvard Pilgrim Healthcare, a Boston-area health plan.^24^ The WLVS and MLVS were designed to investigate the validity of self-reported dietary and other lifestyle assessments over a period of ∼15 months. Women and men who had no history of coronary heart disease, stroke, cancer, or major neurological disease were invited to participate. The study designs have been described in detail elsewhere.^24–26^ The study protocol was approved by the Institutional Review Boards of the Harvard T.H. Chan School of Public Health, the Brigham and Women’s Hospital, and the Harvard Pilgrim Health Care. Informed consents were obtained from the study participants. In this study, 772 women and 691 men who had complete data on at least one 7-day dietary record (7DDR) were included, after excluding two women and one man with implausible total energy intake (>3500 kcal/day for women and >4200 kcal/day for men), respectively.

### Assessments of patterns of coffee drinking timing

NHANES collected dietary data using 24-h dietary recalls; participants reported the timing, types, and amounts of all food and beverages consumed midnight-to-midnight the day before the interview. All participants completed one dietary recall by in-person interview when they underwent physical examination. A second dietary interview was added to the survey starting from 2003, collected 3–10 days later by telephone.^27^ In this study, a single dietary recall was used from 1999 to 2002 and two non-consecutive dietary recalls were used from 2003 onward. Collected dietary recall data were analysed using the US Department of Agriculture (USDA) food composition sources.^27^ Coffee consumption was determined by searching USDA food code descriptions, which included all appropriate beverages containing the term ‘coffee’. Both caffeinated and decaffeinated coffee were selected, and the average amounts of consumption were calculated separately. The coffee drinking timing was categorized in three time periods, including morning (from 4 a.m. to 11:59 a.m.), afternoon (from 12 p.m. to 4:59 p.m.), and evening (from 5 p.m. to 3:59 a.m.). The average frequencies of coffee consumption per day within three periods were calculated, respectively, and were used to define patterns of coffee drinking timing using cluster analysis. Women’s Lifestyle Validation Study and MLVS collected two 7DDRs ∼6 months apart to capture seasonal variability. Participants reported the timing of food consumption and actual intake computed by measuring gram weights of the foods before and after eating. The details of the 7DDR have been reported previously.^25,26^ Seven-day dietary record data were analysed using the USDA food composition sources at the Nutrition Coordinating Center at the University of Minnesota.^28,29^ As done for NHANES, the average frequencies of coffee consumption per day were calculated for three time periods: morning, afternoon, and evening.

To test the robustness of our findings, we (i) repeated the cluster analysis by using the dietary recall for Day 1 or Day 2 only, respectively, as internal validation, (ii) repeated the cluster analysis by using caffeinated or decaffeinated coffee only, respectively, and (iii) performed cluster analyses by using WLVS and MLVS as external validations.

### Assessments of death in National Health and Nutrition Examination Survey

Information on death and death date was identified through linkage to the National Death Index through 31 December 2019. Causes of deaths were classified as CVD with the International Classification of Diseases, Tenth Revision code of I00-I99, and cancer with code of I20-I25. Follow-up time was calculated from the date of interview or examination until the date of death or the end of the study (31 December 2019).

### Statistical analysis

Analyses of covariance (generalized linear models) and χ^2^ tests were used for comparison of continuous and categorical variables, respectively, between participants with different patterns of coffee drinking timing. We used two-step clustering to identify patterns of coffee drinking timing in population.^30–32^ Two-step cluster analysis is a hybrid approach that first performs pre-clustering and is then followed by hierarchical clustering. The optimal number of clusters was estimated by the silhouette width method (the maximum average silhouette width over 2–15 clusters), distances were calculated using log-likelihood, and clustering was performed according to Schwarz’s Bayesian criterion. Details of analysis output are showed in Supplementary material. Hazard ratios (HRs) and 95% confidential intervals (CIs) were estimated in Cox proportional hazards models to evaluate the associations between patterns of coffee drinking timing and coffee intake amounts and the risk of mortality, and follow-up years were used as the underlying time metric. The proportional hazards assumption was tested by the Kaplan–Meier method and Schoenfeld residuals, and no violation was found. Several covariates were adjusted in the models, including age (continuous), sex (male, female), self-reported race and ethnicity (Non-Hispanic White, Non-Hispanic Black, Mexican American, Other Hispanic, and other race), family income [Ratio of family income to poverty <1.3 (low), ≥1.3 and <3.5 (intermediate), and ≥3.5 (high)], educational attainment (less than high school, high school, and some college or above), diabetes (yes, no), hypertension (yes, no), high cholesterol (yes, no), regular physical activity [yes (≥150 min of moderate-intensity activity per week or ≥75 min of vigorous-intensity activity per week or an equivalent combination), no], smoking status [never, former, current (1 to <5 cigarettes, 5 to <10 cigarettes, 10 to <15 cigarettes, 15 to <20 cigarettes, 20 to <25 cigarettes, ≥25 cigarettes per day)], time of smoking cessation among former smokers (<1 year, 1 to <5 years, 5 to <10 years, or ≥10 years before baseline), the Alternative Healthy Eating Index diet score (quintiles), total energy intake (quintiles), caffeinated coffee intake (continuous), decaffeinated coffee intake (continuous), tea intake (continuous), caffeinated soda intake (continuous), short sleep duration [yes (sleep duration <7 h), no] and trouble sleeping (yes, no). Details of the assessment of covariates are described in Supplementary material. We coded missing covariates as a missing indicator category for categorical variables and with mean values for continuous variables. A sensitivity analysis was performed to account for competing risks from other causes for the analyses of CVD-specific and cancer-specific mortality, respectively, by using Fine and Gray’s proportional sub-hazards model.

In addition, we performed stratified analyses by age, sex, family income, smoking status, diagnosed chronic conditions (diagnosed with diabetes, hypertension, or high cholesterol), and short sleep duration. The multiplicative interactions between patterns of coffee drinking timing and these factors were accessed by adding a cross-product term to the models. Moreover, to account for both timing and amounts of coffee consumption simultaneously, we investigated the joint association of patterns of coffee drinking timing and amounts of coffee intake with the risk of mortality.

Statistical analyses were conducted with SAS version 9.4 (SAS Institute Inc. Cary, NC, USA) and SPSS version 23.0 (SPSS, Inc., Chicago, IL, USA). All *P* values were two-sided and *P* < .05 was considered as being statistically significant.

## Results

### Cluster identification and characteristics at baseline

**Figure 1:**
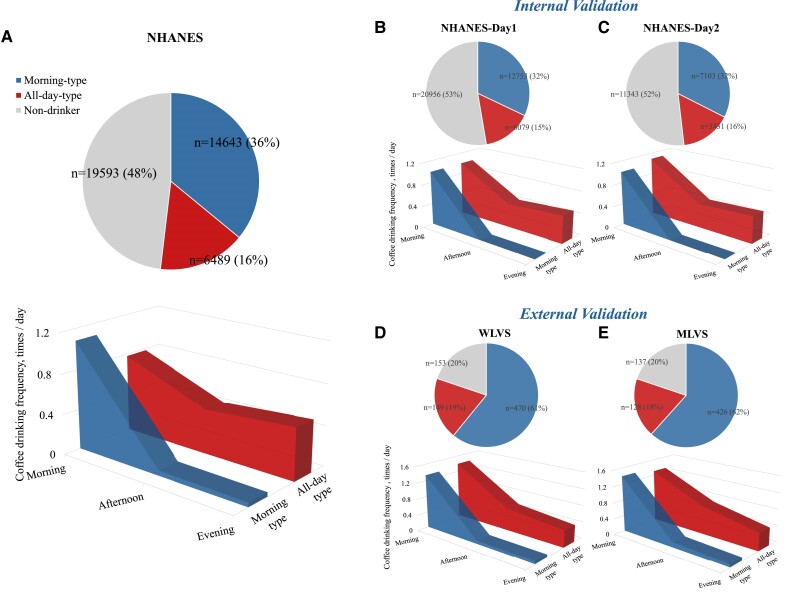
Distribution and characteristics of study participants. (*A*) Distribution of participants according to two-step clustering for the total population in the National Health and Nutrition Examination Survey. (*B*) Distribution of participants provided the first-day dietary data in the National Health and Nutrition Examination Survey. (*C*) Distribution of participants provided the second-day dietary data in the National Health and Nutrition Examination Survey. (*D*) Distribution of participants according to two-step clustering in Women’s Lifestyle Validation Study. (*E*) Distribution of participants according to two-step clustering in Women’s Lifestyle Validation Study. Morning was defined as from 4 a.m. to 11:59 a.m., afternoon from 12 p.m. to 4:59 p.m., and evening from 5 p.m. to 3:59 a.m. NHANES, National Health and Nutrition Examination Survey; WLVS, Women’s Lifestyle Validation Study; MLVS, Momen’s Lifestyle Validation Study

In the present study, two distinct patterns of coffee drinking timing were identified: Cluster 1, morning-type and Cluster 2, all-day-type (*Figure 1A*). Of the 40 725 participants in NHANES, 36% (*n* = 14 643) were identified as having a morning-type pattern, 16% (*n* = 6489) were identified as having an all-day-type pattern, and 48% (*n* = 19 593) were non-coffee drinkers. For participants with morning-type pattern, coffee consumption was mainly concentrated in the morning (between 4 a.m. and 11:59 a.m.) but was barely consumed in the afternoon (between 12 p.m. and 4:59 p.m.) or evening (between 5 p.m. and 3:59 a.m.); for participants with all-day-type pattern, coffee consumption was spread throughout the whole day in the morning, afternoon, and evening. Similar patterns were verified in the following series of sensitivity analyses: if we performed cluster analysis using dietary data from Day 1 or Day 2 only, respectively (*Figure 1B* and *C*); or if we created patterns by using caffeinated or decaffeinated coffee only, respectively (see Supplementary data online, *Figure S2*); or if we repeated the analysis among participants from WLVS and MLVS. Of the 772 women and 691 men from WLVS and MLVS, 61% (*n* = 470) and 62% (*n* = 426) were identified as having a morning-type pattern, 19% (*n* = 149) and 18% (*n* = 128) were identified as having an all-day-type pattern and 20% (*n* = 153) and 20% (*n* = 137) were non-coffee drinkers, respectively (*Figure 1D* and *E*).

The baseline characteristics of participants according to the patterns of coffee drinking timing are presented in *Table 1*. Compared with non-coffee drinkers, participants with morning-type and all-day-type pattern were older, more likely to be White, had higher family income, and a higher prevalence of diabetes, hypertension, and high cholesterol. Among coffee drinkers, participants with morning-type pattern were more likely to consume tea and caffeinated soda but consume less coffee (both caffeinated and decaffeinated coffee) compared with those with all-day patterns.

**Table 1:** Characteristics of participants by patterns of coffee drinking timing in National Health and Nutrition Examination Survey

Characteristics	Non-drinkers	Morning type	All-day-type
*N*	19 593	14 643	6489
Age, years	38.5 ± 17.4	50.6 ± 16.5	51.2 ± 17.6
Female sex, %	9983 (51.0)	7755 (53.0)	3370 (51.9)
Race, %			
Non-Hispanic White	6806 (34.7)	6764 (46.2)	2862 (44.1)
Non-Hispanic Black	5910 (30.2)	2362 (16.1)	664 (10.2)
Mexican American	3698 (18.9)	3002 (20.5)	1386 (21.4)
Other Hispanic	1234 (6.3)	1410 (9.6)	857 (13.2)
Other race	1945 (9.9)	1105 (7.6)	720 (11.1)
Family income,^a^ %			
Low	6444 (32.9)	3531 (24.1)	1812 (27.9)
Intermediate	6580 (33.6)	5146 (35.1)	2267 (34.9)
High	4896 (25.0)	4717 (32.2)	1883 (29.0)
Education levels, %			
Less than high school	4089 (20.9)	3850 (26.3)	1792 (27.6)
High school	4005 (20.4)	3294 (22.5)	1312 (20.2)
Some college or above	8618 (44.0)	7192 (49.1)	3187 (49.1)
Body mass index, kg/m^2^	28.8 ± 7.4	28.7 ± 6.4	28.3 ± 6.1
Diabetes, %	2056 (10.5)	2208 (15.1)	1015 (15.6)
Hypertension, %	5752 (29.4)	6070 (41.5)	2553 (39.3)
High cholesterol, %	3846 (19.6)	4767 (32.6)	2194 (33.8)
Smoking status, %			
Never	11 379 (58.1)	7179 (49.0)	3227 (49.7)
Former	2627 (13.4)	3940 (26.9)	1750 (27.0)
Current	3336 (17.0)	3304 (22.6)	1362 (21.0)
Regular physical activity, %	7704 (39.3)	4966 (34.0)	2141 (33.0)
AHEI diet score	38.8 ± 12.2	41.6 ± 11.8	43.2 ± 11.6
Short sleep duration, %	4811 (34.8)	3655 (34.0)	1705 (34.9)
Trouble sleeping,^b^ %	2887 (20.9)	2734 (25.4)	1082 (22.1)
Total calorie intake, kcal	2063.9 ± 764.7	2000.5 ± 737.5	1979.9 ± 712.6
Tea intake, g	205.7 ± 445.5	177.6 ± 396.5	156.2 ± 354.1
Caffeinated soda intake, g/day	313.9 ± 522.1	239.1 ± 399.5	187.9 ± 350.8
Total coffee intake, g/day			
Mean ± SD	NA	458.5 ± 403.7	597.7 ± 493.5
Median (IQR)	NA	355.2 (296.4)	482.7 (428.35)
Caffeinated coffee intake, g/day	NA	406.7 ± 416.8	514.6 ± 501.7
Decaffeinated coffee intake, g/day	NA	51.8 ± 163.2	83.1 ± 229.8
Percentage of decaffeinated coffee	NA	13.2% ± 32.7	15.1% ± 33.0

Data are presented as mean ± SD, median (IQR), or *n* (%).

AHEI, Alternative Healthy Eating Index; NHANES, National Health and Nutrition Examination Survey.

^a^Family income was defined as a ratio of family income to poverty <1.3 (low), ≥1.3 and <3.5 (intermediate), and ≥3.5 (high).

^b^Short sleep duration was defined as a sleep duration <7 h.

### Association between patterns of coffee drinking timing and the risk of mortality

During a median [interquartile range (IQR)] follow-up of 9.8 (9.1) years, a total of 4295 death events were recorded. Of these death events, 1268 were caused by CVD and 934 were caused by cancer. Compared with non-coffee drinking, a morning-type coffee drinking pattern was significantly associated with a lower risk of all-cause mortality (HR: .88; 95% CI: .81–.96), whereas an all-day-type pattern was not associated with the risk of all-cause mortality (HR: .99; 95% CI: .90–1.10), after adjustment for age, sex, race, NHANES cycles, family income, education levels, diabetes, hypertension, high cholesterol, smoking, physical activity, Alternative Healthy Eating Index score, total calorie intake, and the amount of caffeinated coffee and decaffeinated coffee (*Table 2*). The results did not change if we further adjusted for tea consumed and caffeinated soda consumed (*Table 2*); The observed association remained similar when we further adjusted for short sleep duration and trouble sleeping (HR: .84; 95% CI: .74–.95 for morning-type pattern and HR: .96; 95% CI: .83–1.12 for all-day-type pattern; *Table 2*). We also performed stratified analyses according to age, sex, family income, smoking status, diagnosed chronic conditions, and short sleep duration, and we did not find significant interactions between the patterns of coffee drinking timing and these factors in relation to the risk of all-cause mortality (see Supplementary data online, *Table S1*).

**Table 2:** Association of coffee drinking timing with mortality in National Health and Nutrition Examination Survey

Model	Non-drinker	Morning type	All-day-type
*All-cause mortality*			
Events/total	1484/19 593	1872/14 643	939/6489
Multivariable-adjusted model^a^	1 (reference)	.88 (.81–.96)	.99 (.90–1.10)
Further adjusted for tea and caffeinated soda^b^	1 (reference)	.87 (.80–.95)	.98 (.88–1.09)
Further adjusted for short sleep and trouble sleeping^c^	1 (reference)	.84 (.74–.95)	.96 (.83–1.12)
*CVD-specific mortality*			
Events/total	458/19 593	536/14 643	274/6489
Multivariable-adjusted model^a^	1 (reference)	.81 (.70–.94)	.96 (.79–1.16)
Further adjusted for tea and caffeinated soda^b^	1 (reference)	.80 (.69–.93)	.95 (.78–1.15)
Further adjusted for short sleep and trouble sleeping^c^	1 (reference)	.69 (.55–.87)	.82 (.61–1.10)
*Cancer-specific mortality*			
Events/total	292/19 593	420/14 643	222/6489
Multivariable-adjusted model^a^	1 (reference)	.92 (.77–1.09)	1.05 (.84–1.29)
Further adjusted for tea and caffeinated soda^b^	1 (reference)	.91 (.77–1.08)	1.04 (.84–1.28)
Further adjusted for short sleep and trouble sleeping^cd^	1 (reference)	.97 (.75–1.25)	1.14 (.83–1.56)

CVD, cardiovascular disease; NHANES, National Health and Nutrition Examination Survey.

^a^Models adjusted for age, sex, race and ethnicity, NHANES cycles, family income, education levels, body mass index, diabetes, hypertension, high cholesterol, smoking status, time of smoking cessation, physical activity, Alternative Healthy Eating Index, total calorie intake, caffeinated coffee intake, and decaffeinated coffee intake.

^b^Multivariable-adjusted model + tea intake and caffeinated soda intake.

^c^Multivariable-adjusted model + tea intake and caffeinated soda intake + short sleep duration and trouble sleeping.

^d^Analysis restricted to 29 504 participants for whom the sleep questionnaire was collected from NHANES 2005–18.

For cause-specific mortality, compared with non-coffee drinking, the morning-type pattern was significantly associated with a lower risk of CVD-specific mortality (HR: .69; 95% CI: .55–.87) but not with cancer mortality (HR: .97; 95% CI: .75–1.25). The all-day-type pattern was not associated with risk of CVD-specific mortality or cancer mortality as compared with non-coffee drinking (*Table 2*). The associations were not materially changed if we consider competing risks from other causes for the analyses of CVD-specific and cancer-specific mortality, respectively (see Supplementary data online, *Table S2*). Moreover, we conducted an internal validation of the association between coffee drinking timing and mortality risk by using dietary data from Day 1 and Day 2, respectively, and found similar results (see Supplementary data online, *Table S3*). Similar associations were observed when patterns of caffeinated coffee drinking timing or decaffeinated coffee drinking timing were evaluated separately (see Supplementary data online, *Table S4*).

### Joint association of coffee intake amounts and patterns of coffee drinking timing with mortality risk

We first evaluated the association of coffee intake amounts with mortality in the total study population. We observed that a higher amount of coffee intake was significantly associated with a lower risk of all-cause mortality and CVD-specific mortality (all *P* for trend <.01), but not with cancer-specific mortality (see Supplementary data online, *Table S5*).

**Figure 2:**
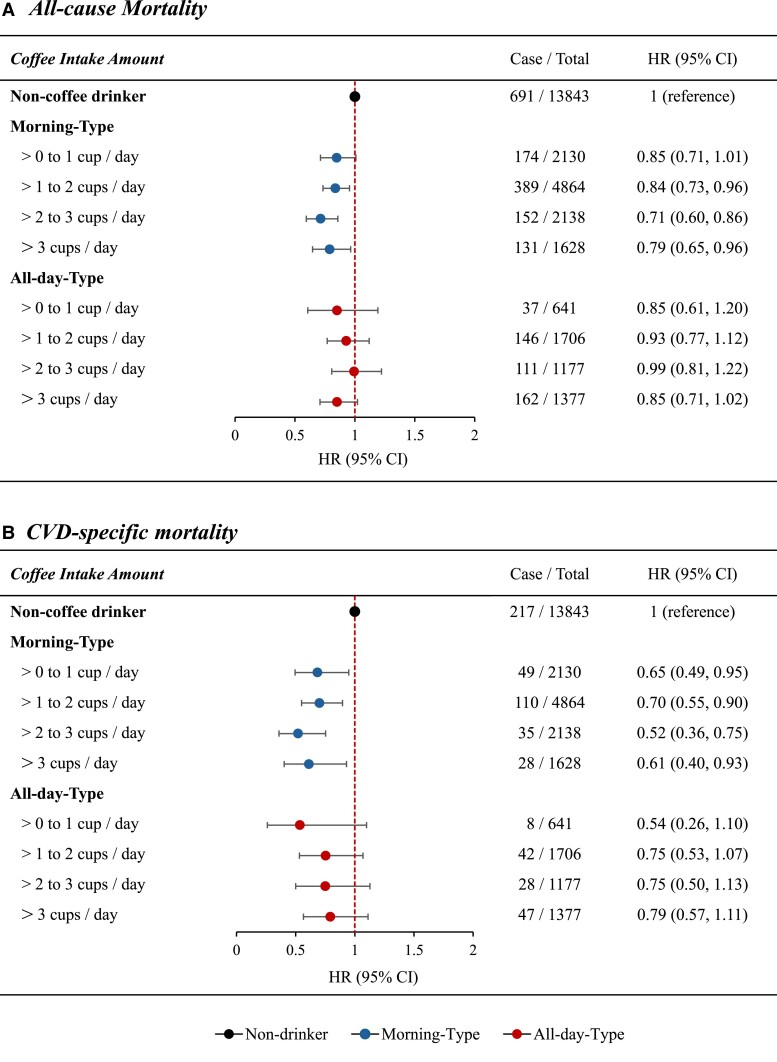
Joint association between coffee intake amounts and coffee drinking timing on the risk of mortality. One cup equal to 8 ounces (1 ounce ~ 28.3 g); models adjusted for age, sex, race, and ethnicity, National Health and Nutrition Examination Survey cycles, family income, education levels, body mass index, diabetes, hypertension, high cholesterol, smoking status, time of smoking cessation, physical activity, Alternative Healthy Eating Index, total calorie intake, tea intake, caffeinated soda intake, percentage of decaf intake, short sleep duration, and trouble sleeping. HR, hazard ratio; CI, confidential interval; CVD, cardiovascular disease

**Figure 3:**
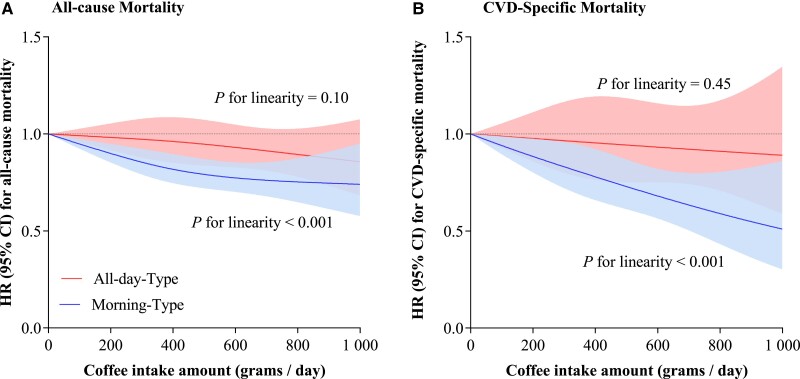
Dose–response relationships between coffee intake amounts and the risk of mortality according to patterns of coffee drinking timing. Models adjusted for age, sex, race, and ethnicity, National Health and Nutrition Examination Survey cycles, family income, education levels, body mass index, diabetes, hypertension, high cholesterol, smoking status, time of smoking cessation, physical activity, Alternative Healthy Eating Index, total calorie intake, tea intake, caffeinated soda intake, percentage of decaf intake, short sleep duration, and trouble sleeping. HR, hazard ratio; CI, confidential interval; CVD, cardiovascular disease

Then, we performed a joint analysis of coffee intake amounts and patterns of coffee drinking timing with mortality risk. The association between coffee intake amounts and the risk of all-cause mortality differed by patterns of coffee drinking timing (*P*-interaction = .031). Compared with non-coffee drinking, both moderate (>1 to 2 cups/day and >2 to 3 cups/day) and heavy (>3 cups/day) coffee consumption were significantly associated with a lower risk of all-cause mortality among participants with morning-type pattern, with an HR (95% CI) of .85 (.71–1.01), .84 (.73–.96), .72 (.60–.86), and .79 (.65–.97) across groups of ‘>0 to 1 cup/day’, ‘>1 to 2 cups/day’, ‘>2 to 3 cups/day’, and ‘>3 cups/day’, respectively (*P* for linear trend <.001), whereas coffee consumption was not significantly associated with all-cause mortality risk among participants with an all-day-type pattern (*Figures 2A* and *3A*). Similar interaction pattern was observed for CVD-specific mortality, but the interaction term was not significant (*Figures 2B* and *3B*).

## Discussion

In this study, we identified two distinct patterns of coffee drinking timing (morning-type and all-day-type) in a nationally representative cohort of US adults and validated these patterns in WLVS and MLVS. Compared with non-coffee drinking, we found that a morning-type coffee drinking pattern was significantly associated with lower risks of all-cause mortality and CVD-specific mortality, independent of the amount of coffee intake. In contrast, an all-day-type pattern was not significantly associated with mortality compared with non-drinking. Moreover, we found that patterns of coffee drinking timing significantly modified the association between coffee intake amounts and all-cause mortality risk, with morning coffee appeared to be more strongly associated with a lower risk of mortality than coffee drinking throughout the day (*Structured Graphical Abstract*).

To our knowledge, this is the first study to investigate the association between patterns of coffee drinking timing and the risk of mortality. Two potential mechanisms could explain our findings. First, consuming coffee in the afternoon or evening may disrupt circadian rhythms. A previous clinical trial showed that heavy coffee consumption in the afternoon or evening was associated with a 30% decrease in peak melatonin production in night-time compared to controls.^33^ Melatonin is a neuroendocrine hormone with a key role in the circadian rhythm, and some evidence suggests that low levels of melatonin are associated with higher oxidative stress levels, blood pressure levels, and CVD risk.^34–36^ Notably, this explanation only applies to caffeinated coffee drinkers. Second, a large portion of coffee's health benefits are achieved through the anti-inflammatory effects of the bioactive substances it contains.^37^ Some pro-inflammatory cytokines and inflammatory markers in the blood also have internal circadian patterns, where they are typically highest in the morning and then gradually decline until reaching their lowest level around 5 p.m.^38,39^ Therefore, when the amounts of coffee intake are similar, the anti-inflammatory effect of a pattern of coffee consumption concentrated in the morning may be more beneficial than that of a pattern of coffee consumption spread across morning, afternoon, and evening. This explanation applies to both caffeinated and decaffeinated coffee consumption. Other mechanisms might also be involved, and future studies are needed to explore the roles of coffee drinking timing in the association of coffee consumption with health outcomes.

Moreover, our findings indicate that coffee consumption was associated with the risk of all-cause mortality differently based on the patterns of coffee drinking timing. Compared with non-coffee drinking, both moderate (> 1 to 2 cups/day and >2 to 3 cups/day) and heavy (>3 cups/day) coffee consumption was significantly associated with a lower risk of all-cause mortality in participants with morning-type pattern, whereas no significant association was observed among those with all-day-type pattern. Further analyses indicate that the observed associations were mainly driven by CVD-specific mortality. Notably, although the inverse association between moderate coffee consumption and mortality risk has been well established in observational studies,^4^ the association between heavy coffee consumption and mortality risk has been inconsistent. In the Nurses’ Health Study and Health Professionals Follow-up study, heavy coffee consumption was not associated with risks of all-cause mortality and CVD-specific mortality.^3,7^ However, in the UK Biobank,^6^ European Prospective Investigation into Cancer and Nutrition study,^40^ and the National Institutes of Health–AARP Diet and Health Study,^5^ even heavy coffee consumption was significantly associated with lower risks of all-cause mortality and CVD-specific mortality. Several hypotheses have been proposed to explain these inconsistent findings. For example, heavy coffee consumption usually correlates with smoking status, thus the observed non-significant inverse association of the heavy coffee consumption with mortality risk in some studies may be attributed to residual confounding by smoking.^3^ Some,^3,5^ but not all,^6,40,41^ previous studies also showed that smoking significantly modified the association of coffee consumption with mortality risk, with heavy coffee consumption significantly associated with a lower risk of all-cause mortality in non-smokers but not in smokers. Other hypotheses suggested that the association of coffee consumption with mortality may be modified by individual differences in genetically determined caffeine metabolism rate, the proportion of decaffeinated consumption, or whether sugar and high saturated fat creamer are added to coffee.^3,6,8,9,11^ However, these hypotheses have not been confirmed by the research to date. The findings of the present study raise the new hypothesis that the association between coffee consumption and mortality risk may differ by the patterns of coffee drinking timing, which may partially explain the inconsistent results for heavy coffee consumption and mortality risk.

The strengths of this study are worth noting. Although the 24-h dietary recall cannot assess long-term food intake, we took advantage of its ability to record the timing of food intake and combined it with cluster analysis to identify patterns of coffee drinking timing in the population. Notably, although the use of cluster analysis to identify latent categories within a population is not novel,^30–32^ to our knowledge, this is the first study to apply cluster analysis to identify patterns of coffee drinking timing. Moreover, to ensure that the identified patterns are stable, this study used the mean of 24-h dietary recall on two non-consecutive days to construct patterns of coffee drinking timing and further validated it in two well-established studies with coffee drinking timing assessed by ‘gold standard’ 7DDR. The present study also has several limitations. First, given the observational nature of the study, the associations observed cannot be interpreted as causality. Second, assessments of exposure and several covariates based on the self-reported questionnaire in NHANES and could be subject to recall bias and measurement errors. Third, although we carefully selected potential confounders in our analysis based on previous literature, the possibility of residual and unmeasured confounding (e.g. shift work and time of getting up) could not be completely ruled out in this analysis. Fourth, although we had carefully adjusted for potential confounding, we could not exclude the possibility that the morning-type coffee drinking pattern is a marker for an overall healthy lifestyle, for example, morning-type coffee drinkers may be more willing to exercise and eat non-ultra-processed foods. Fifth, the association between coffee drinking timing patterns and mortality risk has only been validated internally and external validation is still lacking. Sixth, genetic information was not available in our study, thus we were unable to examine the association between genetically determined caffeine metabolism rate and patterns of coffee drinking timing. Finally, our analyses were performed in the US population, and it is unclear whether our findings can be generalized to other countries with different cultures related to the coffee drinking timing.

## Conclusions

We found that coffee drinking timing was associated with all-cause mortality risk and CVD-specific mortality risk independent of the amounts of coffee intake. Specifically, our findings suggest that coffee drinking in the morning may be more strongly associated with lower mortality than coffee drinking later in the day. Our findings highlight the importance of considering drinking timing in the association between the amounts of coffee intake and health outcomes.

## Supplementary data

Supplementary data are available at *European Heart Journal* online.

## Supplementary Material

ehae871_Supplementary_Data

## Declarations

### Data Availability

The data that support the findings of this study are openly available in NHANES at (https://www.cdc.gov/nchs/nhanes/index.htm). Further information including the procedures to obtain and access data from the Nurses’ Health Studies and Health Professionals Follow-up Study is described at https://www.nurseshealthstudy.org/researchers (contact email: nhsaccess@channing.harvard.edu) and https://sites.sph.harvard.edu/hpfs/for-collaborators/.
