# Supplementary material for: Coffee drinking timing and mortality in US adults
Source: Eur Heart J. 2025 Jan 8;46(8):749–59. doi: 10.1093/eurheartj/ehae871 (PMC11843000; doi:10.1093/eurheartj/ehae871)
Supplement: ehae871_Supplementary_Data [file ehae871_supplementary_data.docx]

**Supplementary Materials**

**Contents**

**SUPPLEMENTARY METHODS**2

Outputs for Two-step cluster analysis2

Assessments of covariates 4

**SUPPLEMENTAL TABLES**6

**Table S1**. Stratified analysis of the association between coffee drinking timing and all-cause mortality in NHANES6

**Table S2**. Association of coffee drinking timing with CVD-specific and cancer-specific mortality after considering competing risk of mortality for other causes in NHANES.7

**Table S3**. Association of coffee drinking timing with mortality by using the Day1 and Day dietary data in NHANES.8

**Table S4**. Association of patterns defined by caffeinated or decaffeinated coffee dinking timing with mortality in NHANES.9

**Table S5**. Association between coffee intake amounts and mortality in total population of NHANES.10

**SUPPLEMENTAL FIGURES**11

**Figure S1**. Flow chart.11

**Figure S2**. Distribution of participants according to patterns defined by caffeinated or decaffeinated coffee dinking timing in NHANES12

## **SUPPLEMENTAL METHODS**

## **Outputs for Two-step cluster analysis**

TWOSTEP CLUSTER

/CONTINUOUS VARIABLES=freq_n1 freq_n2 freq_n3

/DISTANCE LIKELIHOOD

/NUMCLUSTERS AUTO 15 BIC

/HANDLENOISE 0

/MEMALLOCATE 64

/CRITERIA INITHRESHOLD(0) MXBRANCH(8) MXLEVEL(3)

/VIEWMODEL DISPLAY=YES

/PRINT IC COUNT SUMMARY

/SAVE VARIABLE=TSC_67.

**TwoStep Cluster**

| **Auto-Clustering** | | | | |
| --- | --- | --- | --- | --- |
| Number of Clusters | Schwarz's Bayesian Criterion (BIC) | BIC Change^a^ | Ratio of BIC Changes^b^ | Ratio of Distance Measures^c^ |
| 1 | 44001.010 |  |  |  |
| 2 | 26759.285 | -17241.725 | 1.000 | 2.578 |
| 3 | 20108.432 | -6650.853 | .386 | 1.545 |
| 4 | 15825.933 | -4282.499 | .248 | 1.925 |
| 5 | 13630.140 | -2195.793 | .127 | 1.127 |
| 6 | 11688.397 | -1941.743 | .113 | 1.111 |
| 7 | 9946.724 | -1741.673 | .101 | 1.831 |
| 8 | 9022.829 | -923.895 | .054 | 1.099 |
| 9 | 8187.388 | -835.441 | .048 | 1.066 |
| 10 | 7407.166 | -780.222 | .045 | 1.250 |
| 11 | 6794.942 | -612.224 | .036 | 1.109 |
| 12 | 6248.555 | -546.386 | .032 | 1.011 |
| 13 | 5708.970 | -539.586 | .031 | 1.062 |
| 14 | 5204.551 | -504.419 | .029 | 1.239 |
| 15 | 4808.946 | -395.605 | .023 | 1.290 |

| a. The changes are from the previous number of clusters in the table. |
| --- |
| b. The ratios of changes are relative to the change for the two cluster solution. |
| c. The ratios of distance measures are based on the current number of clusters against the previous number of clusters. |

| **Cluster Distribution** | | | | |
| --- | --- | --- | --- | --- |
|  | | N | % of Combined | % of Total |
| Cluster | 1 | 14643 | 69.3% | 36.0% |
|  | 2 | 6489 | 30.7% | 15.9% |
|  | Combined | 21132 | 100.0% | 51.9% |
| Excluded Cases | | 19593 |  | 48.1% |
| Total | | 40725 |  | 100.0% |


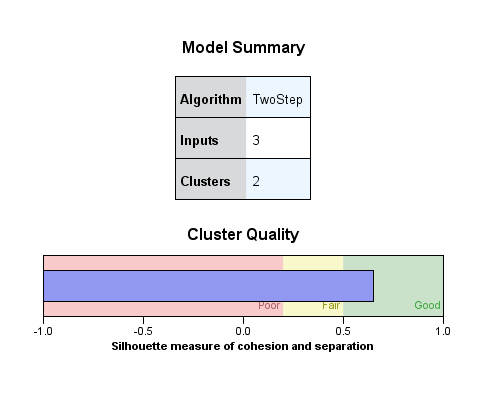
**Cluster Profiles**

| **Centroids** | | | | | | |  |
| --- | --- | --- | --- | --- | --- | --- | --- |
|  | | Mean | | Mean | | Mean | |
|  |  | Mean | Std. Deviation | Mean | Std. Deviation | Mean | Std. Deviation |
| Cluster | 1 | 1.082 | .2427 | .005 | .0521 | .004 | .0438 |
|  | 2 | .781 | .6109 | .447 | .4758 | .518 | .4981 |
|  | Combined | .990 | .4181 | .141 | .3360 | .162 | .3657 |

The maximum average silhouette width is 0.7

## **Assessments of** **covariates**

In NHANES, the self-reported covariates collected by standardized questionnaires included age, sex, race and ethnicity (Non-Hispanic White [hereafter White], Non-Hispanic Black [hereafter Black], Mexican American, Other Hispanic, and other race), family income (Ratio of family income to poverty < 1.3 [low], ≥ 1.3 and < 3.5 [intermediate], and ≥ 3.5 [high]), educational attainment (less than high school, high school, and some college or above), physical activity (minutes of moderate or vigorous physical activity per week), smoking status (never, former [quitting < 1 year, 1 to < 5 years, 5 to < 10 years, or ≥ 10 years before baseline], current [1 to < 5 cigarettes, 5 to < 15 cigarettes, 15 to < 25 cigarettes, 25 to < 35 cigarettes, 35 to < 45 cigarettes, ≥ 45 cigarettes per day]), time of smoking cessation among former smokers (<1 year, 1 to <5 years, 5 to <10 years, or ≥10 years before baseline) short sleep (sleep duration < 7 hours) and trouble sleeping (yes, no). The Alternative Healthy Eating Index (AHEI) diet score (without considering alcohol intake), total calorie intake, caffeinated coffee intake, decaffeinated coffee intake, tea intake, total coffee intake and caffeinated soda intake were estimated by information collected from 24-h dietary recall.

Weight, standing height, blood pressure, plasma levels of glucose, glycated hemoglobin (HbA1c) were measured in mobile examination centers with standard protocols. Body mass index (BMI) was calculated as weight in kilograms divided by standing height in meters squared. Diabetes was defined as a self-reported history of diabetes or a fasting plasma glucose ≥126 mg/dL or HbA1c levels ≥ 6.5% or taking antidiabetic medications. Hypertension was defined as a self-reported history of hypertension or a systolic blood pressure ≥ 140 mmHg or a diastolic blood pressure ≥ 90 mm Hg or taking antihypertensive medications. High cholesterol was defined as a self-reported history of high cholesterol or taking lipid-lowering medications.

**SUPPLEMENTARY TABLES**

**Table S1.** Stratified analysis of the association between coffee drinking timing and all-cause mortality in NHANES.

|  | **Non-drinker** | **Morning type** | **All-day type** | ***P* for interaction** |
| --- | --- | --- | --- | --- |
| **Age** |  |  |  | 0.07 |
| <45 years old | 1 (reference) | 0.76 (0.57, 1.00) | 0.74 (0.49, 1.10) |  |
| ≥45 years old | 1 (reference) | 0.91 (0.83, 0.99) | 1.02 (0.92, 1.14) |  |
| **Sex** |  |  |  | 0.90 |
| Women | 1 (reference) | 0.84 (0.70, 1.01) | 1.04 (0.83, 1.32) |  |
| Men | 1 (reference) | 0.86 (0.73, 1.02) | 0.95 (0.77, 1.17) |  |
| **Family income** |  |  |  | 0.18 |
| Low | 1 (reference) | 1.02 (0.88, 1.17) | 1.17 (0.98, 1.39) |  |
| Medium | 1 (reference) | 0.77 (0.68, 0.88) | 0.92 (0.78, 1.08) |  |
| High | 1 (reference) | 0.85 (0.70, 1.02) | 0.84 (0.65, 1.09) |  |
| **Smoking status** |  |  |  | 0.09 |
| Never | 1 (reference) | 0.94 (0.82, 1.08) | 1.04 (0.87, 1.25) |  |
| Former | 1 (reference) | 0.92 (0.79, 1.07) | 1.20 (1.00, 1.44) |  |
| Current (≤ 10 cigarettes/day) | 1 (reference) | 0.70 (0.55, 0.91) | 0.72 (0.51, 1.02) |  |
| Current (> 10 cigarettes/day) | 1 (reference) | 0.81 (0.64, 1.02) | 0.81 (0.60, 1.09) |  |
| **Diagnosed chronic conditions** |  |  |  | 0.71 |
| Yes | 1 (reference) | 0.91 (0.82, 1.00) | 1.04 (0.91, 1.18) |  |
| No | 1 (reference) | 0.76 (0.63, 0.91) | 0.85 (0.66, 1.08) |  |
| **Short sleep duration** |  |  |  | 0.16 |
| Yes | 1 (reference) | 0.97 (0.79, 1.18) | 1.01 (0.79, 1.31) |  |
| No | 1 (reference) | 0.79 (0.68, 0.92) | 0.96 (0.79, 1.16) |  |

Models adjusted for age, sex, race, NHANES cycles, family income, education levels, body mass index, diabetes, hypertension, high cholesterol, smoking status, time of smoking cessation, physical activity, Alternative Healthy Eating Index, total calorie intake, caffeinated coffee intake and decaffeinated coffee intake. NHANES, National Health and Nutrition Examination Survey.

**Table S2.** Association of coffee drinking timing with CVD-specific and cancer-specific mortality after considering competing risk of mortality for other causes in NHANES.

| **Model** | **Timing pattern** | | |
| --- | --- | --- | --- |
|  | ***Non-drinker*** | ***Morning type*** | ***All-day type*** |
| **CVD-specific Mortality** |  | | |
| Multivariable adjusted model ^a^ | 1 (reference) | 0.81 (0.70, 0.94) | 0.90 (0.74, 1.10) |
| Further adjusted for tea and caffeinated soda ^b^ | 1 (reference) | 0.81 (0.69, 0.94) | 0.90 (0.74, 1.10) |
| Further adjusted for short sleep and trouble sleeping ^c, d^ | 1 (reference) | 0.70 (0.56, 0.87) | 0.79 (0.59, 1.05) |
| **Cancer-specific Mortality** |  |  |  |
| Multivariable adjusted model ^a^ | 1 (reference) | 0.94 (0.80, 1.12) | 1.04 (0.84, 1.29) |
| Further adjusted for tea and caffeinated soda ^b^ | 1 (reference) | 0.94 (0.79, 1.12) | 1.03 (0.84, 1.28) |
| Further adjusted for short sleep and trouble sleeping ^c, d^ | 1 (reference) | 1.01 (0.78, 1.29) | 1.15 (0.83, 1.59) |

^a^, Models adjusted for age, sex, race and ethnicity, NHANES cycles, family income, education levels, body mass index, diabetes, hypertension, high cholesterol, smoking status, time of smoking cessation, physical activity, Alternative Healthy Eating Index, total calorie intake, caffeinated coffee intake and decaffeinated coffee intake.

^b^, Multivariable adjusted model + tea intake and caffeinated soda intake.

^c^, Multivariable adjusted model + tea intake and caffeinated soda intake + short sleep duration and trouble sleeping.

^d^, Analysis restricted to 29,504 participants for whom the sleep questionnaire was collected from NHANES 2005-2018.

NHANES, National Health and Nutrition Examination Survey. CVD, cardiovascular disease.

**Table S3.** Association of coffee drinking timing with mortality by using the Day1 and Day dietary data in NHANES.

| **Model** | **Non-drinker** | **Morning type** | **All-day type** |
| --- | --- | --- | --- |
| ***All-cause mortality*** |  |  |  |
| Day 1 | 1 (reference) | 0.89 (0.83, 0.97) | 0.93 (0.84, 1.03) |
| Day 2 | 1 (reference) | 0.87 (0.80, 0.94) | 0.95 (0.86, 1.05) |
| ***CVD-specific Mortality*** |  | | |
| Day 1 | 1 (reference) | 0.86 (0.75, 0.99) | 0.86 (0.71, 1.04) |
| Day 2 | 1 (reference) | 0.81 (0.69, 0.94) | 0.93 (0.77, 1.13) |
| ***Cancer-specific Mortality*** |  | | |
| Day 1 | 1 (reference) | 0.99 (0.84, 1.17) | 1.09 (0.89, 1.34) |
| Day 2 | 1 (reference) | 0.95 (0.80, 1.12) | 1.07 (0.87, 1.32) |

^a^, Models adjusted for age, sex, race and ethnicity, NHANES cycles, family income, education levels, body mass index, diabetes, hypertension, high cholesterol, smoking status, time of smoking cessation, physical activity, Alternative Healthy Eating Index, total calorie intake, caffeinated coffee intake and decaffeinated coffee intake.

NHANES, National Health and Nutrition Examination Survey. CVD, cardiovascular disease.

**Table S4.** Association of patterns defined by caffeinated or decaffeinated coffee dinking timing with mortality in NHANES.

| **Model** | **Timing pattern** | | |
| --- | --- | --- | --- |
| ***Caffeinate-coffee drinking timing pattern*** | ***Non-drinker*** | ***Morning type*** | ***All-day type*** |
| **All-cause mortality** |  |  |  |
| Multivariable adjusted model ^a^ | 1 (reference) | 0.91 (0.84, 0.98) | 0.96 (0.86, 1.07) |
| Further adjusted for tea and caffeinated soda ^b^ | 1 (reference) | 0.90 (0.83, 0.98) | 0.95 (0.85, 1.06) |
| Further adjusted for short sleep and trouble sleeping ^c, d^ | 1 (reference) | 0.89 (0.79, 0.99) | 0.97 (0.83, 1.14) |
| **CVD-specific Mortality** |  | | |
| Multivariable adjusted model ^a^ | 1 (reference) | 0.85 (0.73, 0.99) | 0.95 (0.77, 1.16) |
| Further adjusted for tea and caffeinated soda ^b^ | 1 (reference) | 0.85 (0.73, 0.99) | 0.94 (0.76, 1.15) |
| Further adjusted for short sleep and trouble sleeping ^c, d^ | 1 (reference) | 0.75 (0.60, 0.95) | 0.87 (0.64, 1.19) |
| **Cancer-specific Mortality** |  |  |  |
| Multivariable adjusted model ^a^ | 1 (reference) | 1.02 (0.86, 1.20) | 1.13 (0.91, 1.41) |
| Further adjusted for tea and caffeinated soda ^b^ | 1 (reference) | 1.01 (0.85, 1.20) | 1.13 (0.91, 1.40) |
| Further adjusted for short sleep and trouble sleeping ^c, d^ | 1 (reference) | 1.11 (0.86, 1.42) | 1.28 (0.94, 1.76) |
| ***Decaffeinate-coffee drinking timing pattern*** | ***Non-drinker*** | ***Morning type*** | ***All-day type*** |
| **All-cause mortality** |  |  |  |
| Multivariable adjusted model ^a^ | 1 (reference) | 0.87 (0.77, 0.99) | 1.21 (1.02, 1.43) |
| Further adjusted for tea and caffeinated soda ^b^ | 1 (reference) | 0.87 (0.76, 0.99) | 1.20 (1.01, 1.42) |
| Further adjusted for short sleep and trouble sleeping ^c, d^ | 1 (reference) | 0.79 (0.65, 0.97) | 1.21 (0.93, 1.58) |
| **CVD-specific Mortality** |  |  |  |
| Multivariable adjusted model ^a^ | 1 (reference) | 0.74 (0.59, 0.94) | 0.92 (0.67, 1.26) |
| Further adjusted for tea and caffeinated soda ^b^ | 1 (reference) | 0.74 (0.59, 0.94) | 0.92 (0.67, 1.25) |
| Further adjusted for short sleep and trouble sleeping ^c, d^ | 1 (reference) | 0.65 (0.45, 0.92) | 0.66 (0.39, 1.13) |
| **Cancer-specific Mortality** |  |  |  |
| Multivariable adjusted model ^a^ | 1 (reference) | 0.86 (0.65, 1.14) | 1.36 (0.96, 1.93) |
| Further adjusted for tea and caffeinated soda ^b^ | 1 (reference) | 0.86 (0.65, 1.14) | 1.36 (0.96, 1.92) |
| Further adjusted for short sleep and trouble sleeping ^c, d^ | 1 (reference) | 0.82 (0.54, 1.26) | 1.74 (1.06, 2.88) |

^a^, Models adjusted for age, sex, race and ethnicity, NHANES cycles, family income, education levels, body mass index, diabetes, hypertension, high cholesterol, smoking status, time of smoking cessation, physical activity, Alternative Healthy Eating Index, total calorie intake, caffeinated coffee intake and decaffeinated coffee intake.

^b^, Multivariable adjusted model + tea intake and caffeinated soda intake.

^c^, Multivariable adjusted model + tea intake and caffeinated soda intake + short sleep duration and trouble sleeping.

^d^, Analysis restricted to 29,504 participants for whom the sleep questionnaire was collected from NHANES 2005-2018.

NHANES, National Health and Nutrition Examination Survey. CVD, cardiovascular disease.

**Table S5.** Association between coffee intake amounts and mortality in total population of NHANES.

| Outcomes | Non-drinker | Coffee intake amount | | | | *P* for trend |
| --- | --- | --- | --- | --- | --- | --- |
|  |  | > 0 to 1 cup | > 1 to 2 cups | > 2 to 3 cups | > 3 cups |  |
| All-cause Mortality | 1 (reference) | **0.84 (0.72, 0.99)** | **0.86 (0.76, 0.97)** | **0.81 (0.70, 0.94)** | **0.82 (0.71, 0.96)** | **0.003** |
| CVD-specific Mortality | 1 (reference) | **0.66 (0.48, 0.89)** | **0.71 (0.57, 0.89)** | **0.60 (0.45, 0.81)** | **0.72(0.54, 0.96)** | **0.002** |
| Cancer-specific Mortality | 1 (reference) | 1.00 (0.70, 1.43) | 1.03 (0.79, 1.34) | 1.08 (0.79, 1.47) | 1.18 (0.87, 1.61) | 0.30 |

Models adjusted for age, sex, race, NHANES cycles, family income, education levels, body mass index, type 2 diabetes, hypertension, high cholesterol, smoking status, time of smoking cessation, physical activity, Alternative Healthy Eating Index, total calorie intake, percentage of decaffeinated coffee intake, tea intake, caffeinated soda intake, short sleep duration and trouble sleeping.

NHANES, National Health and Nutrition Examination Surve; CVD, cardiovascular disease.

**SUPPLEMENTARY FIGURE**

**Figure S1.** Flow chart.

**
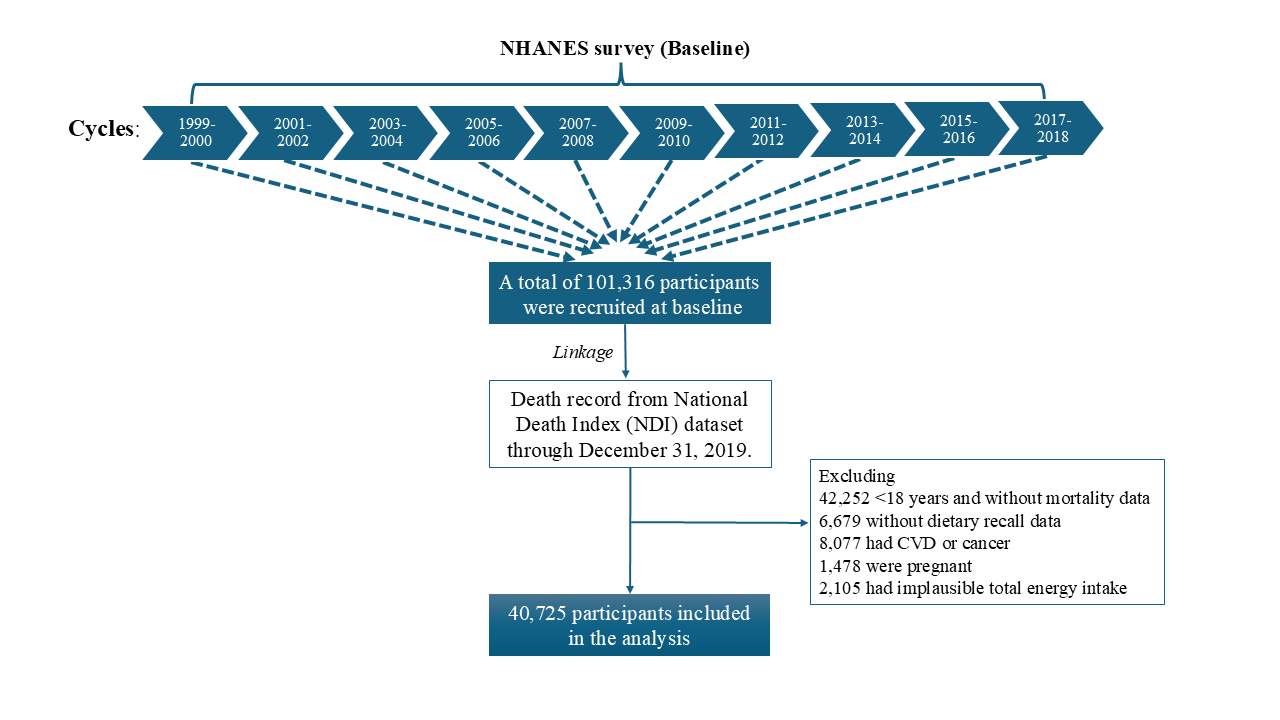
**

**Figure S2.** Distribution of participants according to patterns defined by caffeinated or decaffeinated coffee dinking timing in NHANES.


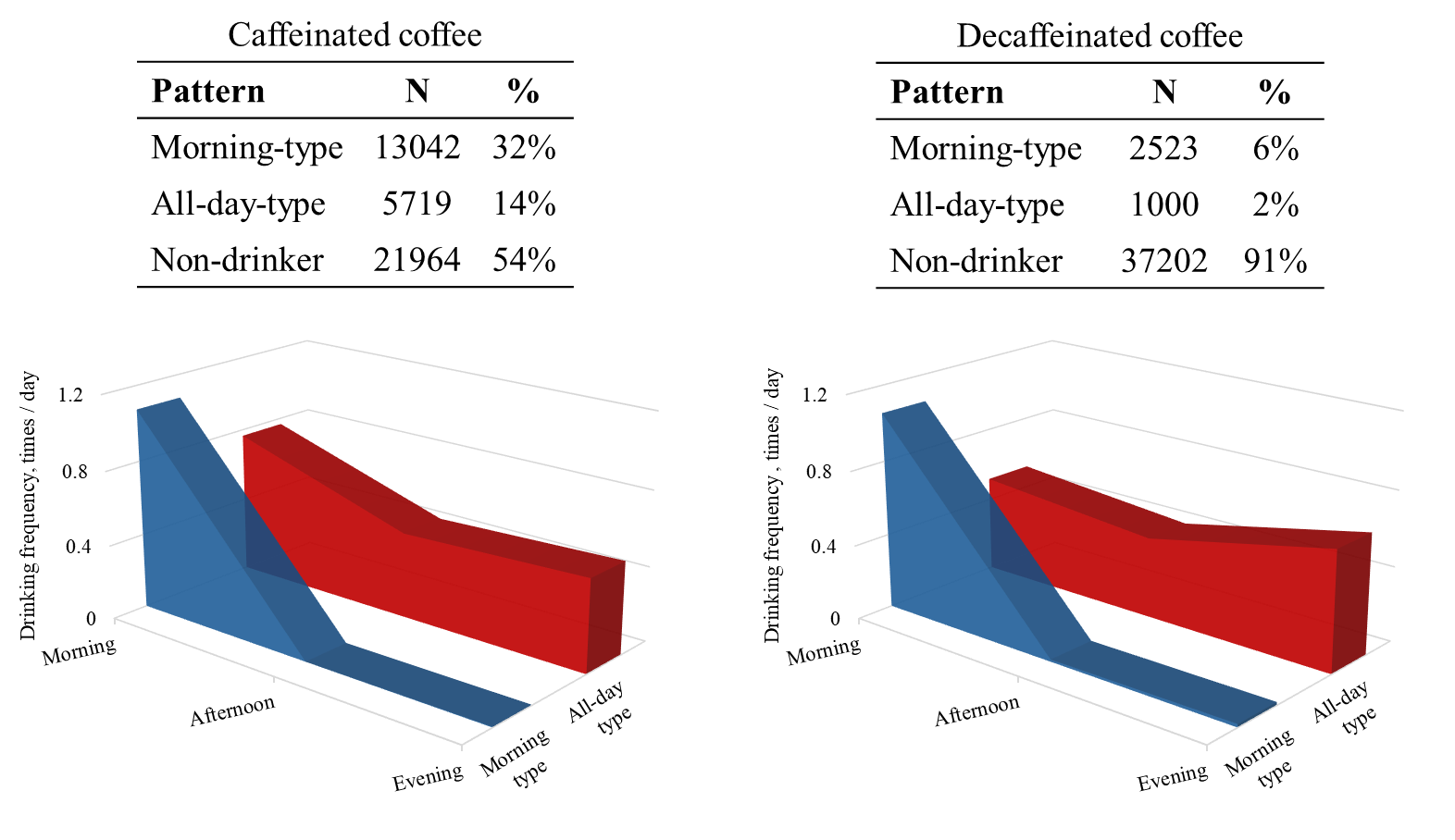


Morning was defined as from 4:00 am. to 11:59 am, afternoon from 12:00 pm. to 4:59 pm and evening from 5:00 pm. to 3:59 am. NHANES, National Health and Nutrition Examination Survey.
